# Myelodysplastic Syndrome (MDS) With *KMT2A::CBL* Rapidly Progressed to Acute Myeloid Leukaemia (AML)

**DOI:** 10.1002/jha2.70260

**Published:** 2026-03-09

**Authors:** Ke Xu, Temenuzhka Boneva, Elisabeth Nacheva

**Affiliations:** ^1^ Department of Haematology University College London Hospitals NHS Foundation Trust London UK; ^2^ Specialist Integrated Haematology Malignancy Diagnostic Service Health Services Laboratories University College London Hospitals NHS Foundation Trust London UK; ^3^ UCL School of Life and Medical Sciences University College London London UK

1

A 73‐year‐old female presented with progressive isolated normocytic anaemia. The blood work showed haemoglobin 95 g/L, white blood cells 4.7 × 10^9^/L (neutrophil 2.6 × 10^9^/L, monocytes 0.4 × 10^9^/L) and platelets 172 × 10^9^/L. The bone marrow aspirate showed dysplastic neutrophils (> 10%) and erythroblasts (> 10%), 22% monocytes and 2% of blasts (Figure 1A). Flow cytometry showed 0.1% blasts and 57% CD14+CD56+ monocytes. Iron stain showed 15% ring sideroblasts (Figure 1A). Targeted fluorescence in situ hybridisation (FISH) analysis (Cytocell) and chromosomal microarray analysis (CMA) using 8 × 60 K oligonucleotide arrays (Agilent) showed loss of the 7q22.1 region (housing *CUX1* gene among others) and a 765 bp deletion within 11q23.3, suggestive of *KMT2A::CBL* fusion gene formation (Figure 1B). RNA sequencing (Archer Fusion Plex Pan‐Heme panel) showed *KMT2A::CBL* fusion transcript. Pan‐Heme VariantPlex NGS (157 gene panel) showed *TET2* p.Leu500Ile (VAF 81%), *DIS3* p.Tyr867Ter (VAF 51%) and *SRSF2* p.Pro95Arg (VAF 47%) variants. The patient was diagnosed with MDS, multiple lineage dysplasia, IPSS‐R intermediate risk and IPSS‐M moderate high risk. MDS with ring sideroblast usually has a better outcome, but the presence of *KMT2A::CBL* puts this patient at high risk of progression to AML. She was monitored closely. Two and a half months later, she developed worsening anaemia, mild neutropenia and monocytosis. Hb 80 g/L, WBC 4.1 × 10^9^/L (neutrophils 1.3 × 10^9^/L, monocytes 1.5 × 10^9^/L) and platelets 157 × 10^9^/L. The repeat bone marrow aspirate showed 50% monocytoid blasts on the background of MDS (Figure 1C). By flow cytometry, the blasts were positive for CD33, HLADR, CD15, CD56, CD11b, cMPO and negative for CD34, CD117, CD19, CD22, cCD79a and cCD3. Myeloid NGS showed *SRSF2* p.Pro95Arg (VAF 48%), *TET2* p.Leu500IlefsTer4 (VAF 83%) and *TET2* p.Gly1815GlufsTer7 (VAF 4%) variants. Targeted FISH and CMA again showed loss of the *CUX1* gene, 12q24 loss and a cryptic deletion within 11q23.3. RNAseq showed persistence of *KMT2A::CBL* fusion. She was diagnosed with AML with *KMT2A* rearrangement (*KMT2Ar*).

**FIGURE 1:**
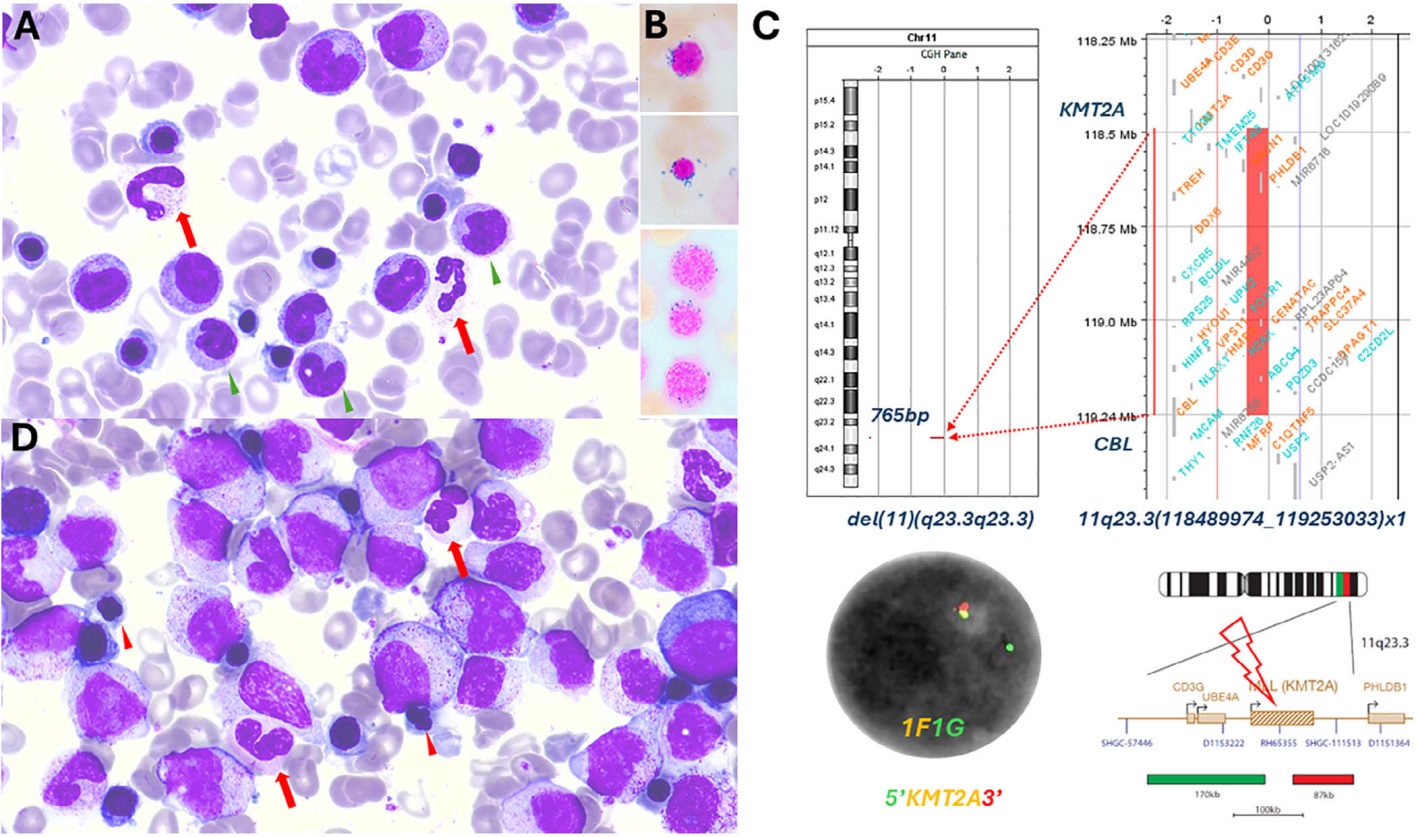
(A) Bone marrow aspirate (May‐Grünwald–Giemsa stain, ×100 objective) at diagnosis showed > 10% dysplastic hypogranulated neutrophils (red arrow), monocytosis (green arrow head) and no excess of blasts; (B) Iron stain (×100 objective) showed ring sideroblasts. (C) At diagnosis, FISH (DAPI staining, ×100 objective) using *KMT2A* break‐apart probe showed 1F1G signal, suggestive of del(11). CMA showed a cryptic deletion within 11q23.3, suggestive of *KMT2A::CBL* fusion. (D) Bone marrow aspirate (May‐Grünwald–Giemsa stain, ×100 objective) at disease progression showed large monocytoid blasts with open chromatin, prominent nucleoli, no auer rods and dysplastic erythroblasts (red arrow head showing irregular nuclear contour) and dysplastic neutrophils (red arrows showing Pseudo‐Pelger–Huët forms).

Here, we report a rare case of MDS with *KMT2A::CBL* and low blast count that rapidly progressed to *KMT2Ar* AML. *KMT2A* rearrangement is considered to be AML‐defining by WHO5 [1] and ICC classification [2]. WHO5 requires an excess of myeloblasts in the bone marrow (≥ 5%) or peripheral blood. ICC requires myeloblast *≥ *10%. *KMT2A* rearrangement is rare in MDS. Arber DA et al. reported 31 *KMT2Ar* MDS/chronic myelomonocytic leukaemia (CMML) cases with similar median overall survival compared to the 100 cases of *KMT2Ar* AML (12 months vs. 14.4 months, *p *= 0.62), raising the hypothesis that *KMT2Ar* MDS/CMML and AML likely represent the same biologic entity [3]. Given the grey zone for MDS with *KMT2A* rearrangement without blast excess, a multidisciplinary team approach is recommended for optimal management on a case‐by‐case basis.

## Author Contributions

K.X. wrote up the manuscript. K.X., T.B. and E.N. critically revised the final version of the manuscript.

## Funding

The authors have nothing to report.

## Ethics Statement

The authors have nothing to report.

## Consent

The authors have nothing to report.

## Conflicts of Interest

The authors declare no conflicts of interest.

## Data Availability

The data that support the findings of this study are available from the corresponding author upon reasonable request.
